# Emerging functional similarities and divergences between *Drosophila* Spargel/dPGC-1 and mammalian PGC-1 protein

**DOI:** 10.3389/fgene.2014.00216

**Published:** 2014-07-10

**Authors:** Subhas Mukherjee, Mohammed A. Basar, Claudette Davis, Atanu Duttaroy

**Affiliations:** ^1^Department of Biology, Howard UniversityWashington, DC, USA; ^2^Biology Undergraduate Program, College of Science, George Mason UniversityFairfax, VA, USA

**Keywords:** *Drosophila*, PGC-1, mitochondria, oogenesis, RNA processing

## Abstract

Peroxisome Proliferator Activated Receptor Gamma Co-activator-1 (PGC-1) is a well-conserved protein among all chordates. Entire *Drosophila* species subgroup carries a *PGC-1* homolog in their genome called *spargel/dPGC-1* showing very little divergence. Recent studies have reported that significant functional similarities are shared between vertebrate and invertebrate PGC-1's based on their role in mitochondrial functions and biogenesis, gluconeogenesis, and most likely in transcription and RNA processing. With the help of genetic epistasis analysis, we established that *Drosophila* Spargel/dPGC-1 affects cell growth process as a terminal effector in the Insulin-TOR signaling pathway. The association between Spargel/dPGC-1 and Insulin signaling could also explain its role in the aging process. Here we provided a further comparison between Spargel/dPGC-1 and PGC-1 focusing on nuclear localization, oxidative stress resistance, and a possible role of Spargel/dPGC-1 in oogenesis reminiscing the role of Spargel in reproductive aging like many Insulin signaling partners. This led us to hypothesize that the discovery of newer biological functions in *Drosophila* Spargel/dPGC-1 will pave the way to uncover novel functional equivalents in mammals.

Homeothermic mammals utilize the Peroxisome Proliferator Activated Receptor Gamma Co-activator 1 (PGC-1) as a thermogenic regulator (maintains body temperature) to protect against excessive cold or excess calorie intake (Puigserver et al., 1998). Thus PGC-1 is expressed in tissues with high metabolic requirement and it is linked to multiple metabolic pathways such as gluconeogenesis, adipogenesis, myogenesis, and mitogenesis (Handschin and Spiegelman, 2006). Apart from metabolism, PGC-1 might play a central role in maintaining oxidative homeostasis (Austin and St-Pierre, 2012). Homologs of *PGC-1* were found in all chordates including the fish genomes (Lin et al., 2005) where body temperature regulation isn't necessary (with the exception of a few marine species). Among invertebrate models, only *Drosophila* carries a single *PGC-1* homolog in its genome (Gershman et al., 2007) whereas other invertebrate models such as yeast and *C. elegans* do not carry any *PGC-1* homologous sequence (Lin et al., 2005). *Drosophila PGC-1*, designated as *spargel/dPGC-1* (Tiefenbock et al., 2010), is well conserved in distantly related *Drosophila* species subgroups with its C-terminal RS and RRM domains (Figure 1A). Two notable differences between PGC-1 and Spargel/dPGC-1 are: the C-terminal FDSLL domain of PGC-1 is replaced with FEALL in all *Drosophila* species (Gershman et al., 2007; Tiefenbock et al., 2010; Bugger et al., 2011), and the larger size of Spargel/dPGC-1 protein as it carries ~300 more amino acids than PGC-1 (Figure 1A). Although nuclear receptors are generally known to interact with the leucine rich motifs (LXXLL) (Matsuda et al., 2004), the FEALLL variant of this motif in *Drosophila* is able to interact with the nuclear receptors as well (Wang et al., 2007). In light of the fact that significant functional overlap exists between the three PGC-1 homologs in mice PGC-1α, PGC-1β and PRC, which makes it difficult to tease apart their relative roles *in vivo*, we propose that the presence of a single *Drosophila PGC-1* homolog will provide an enormous advantage to study the function of this essential transcriptional coactivator in an alternate model. Within the last few years, significant functional homologies have surfaced between mammalian PGC-1 and *Drosophila* Spargel/dPGC-1, which called for a discussion of this topic in greater detail.

**Figure 1:**
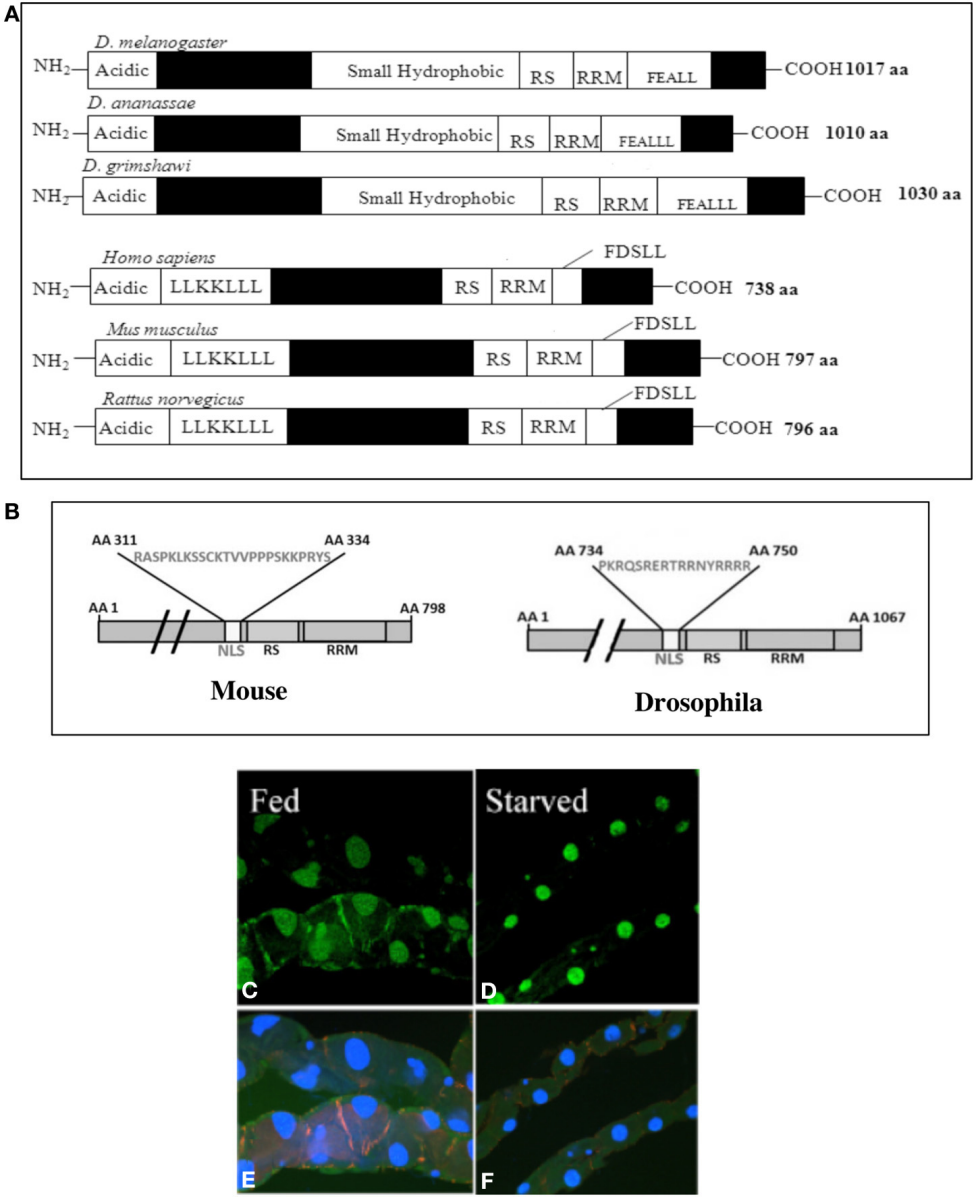
**(A)** Comparison of the Spargel/dPGC-1 protein structure in 3 widely divergent subgroups of *Drosophila melanogaster*, *D. ananassae*, and *D. grimshawi* (van der Linde and Houle, 2008). Vertebrate PGC-1 is about 300 amino acids shorter in length than the Spargel/dPGC-1. Filled boxes represent regions of non-homology. **(B)** An authentic Nuclear Localization Signal (NLS) has been found in mammalian PGC-1 with the help of NLS Predict Software. **(C)** Nuclear localization of Spargel is documented in the gut tissue with a Spargel-GFP protein (green). Following 48 h of starvation, the gut turns thinner and Insulin signaling is reduced, but it imposes no effect on nuclear localization of the Spargel-GFP protein **(D)**. **(E,F)** DAPI staining of the same gut tissue.

## Energy metabolism

As a transcriptional coactivator PGC-1 activates many nuclear receptor, which in turn regulate the transcription activity of variety of nuclear genes (Puigserver and Spiegelman, 2003). Similarly, Spargel/dPGC-1 regulates the expression of mitochondrial oxidative phosphorylation (OXPHOS) genes through *NRF1* (Nuclear Respiratory Factor) homolog *delg* (Tiefenbock et al., 2010). *Spargel/dPGC-1* gain of function (overexpression) correlates with an increased rate of mitochondrial oxygen consumption (Rera et al., 2011) and ATP production (Mukherjee and Duttaroy, 2013), enhanced mitochondrial DNA content, increased enzyme activity and protein production in the mitochondrial matrix (Rera et al., 2011). These observations are reminiscent of the effect of PGC-1α on mitochondrial biogenesis, functional capacity and energy metabolism (Liu and Lin, 2011). Thus, regulation of mitochondrial function is truly an ancestral function for PGC-1 group of proteins.

## Intracellular localization

Serine-Arginine (SR) repeats and RNA Recognition Motifs (RRM) are classical hallmarks of “RNA processing domains.” Furthermore, localization of PGC-1 in the nuclear compartment where it is concentrated in nuclear speckles along with splicing factor α-U1 and SR splicing factor SC-35 are irrefutable evidence that PGC-1 is involved in the splicing complex (Monsalve et al., 2000). We recently demonstrated that a Spargel-GFP fusion protein also localizes itself in the nucleus forming distinct punctate structures (Mukherjee and Duttaroy, 2013). An authentic Nuclear Localization Signal (NLS) was uncovered in Spargel/dPGC-1 with the help of a NLS predict software (Mukherjee and Duttaroy, 2013) that most likely assists in Spargel/dPGC-1 localization into the nucleus. With the help of the same software we now located a NLS between the amino acids 311–334 in mammalian PGC-1 (score 0.93) (Figure 1B), which should also justify the presence of PGC-1 protein in the nucleus (Monsalve et al., 2000).

An earlier study claimed that activation of Insulin signaling is important for transport of Spargel/dPGC-1 protein from cytoplasm to the nucleus (Tiefenbock et al., 2010) and Spargel/dPGC-1 is now established as a member of the Insulin-TOR signaling pathway (Mukherjee and Duttaroy, 2013). Nutrient availability controls the Insulin/TOR signaling pathway from TOR downstream (Takano et al., 2001) therefore, under starvation condition, Insulin mediated signal transduction is reduced. We rationalized that if Insulin signaling is essential for nuclear localization of Spargel/dPGC-1 then reduction of this signal should sequester the protein to the cytosol. To test this prediction, gut malphigian tubule preparations were obtained from flies following two days of starvation (only water given to prevent dehydration) and analyzed for Spargel expression. Since the localization of Spargel/dPGC-1 is still restricted to the nucleus following starvation (Figures 1C–F) this supports that cellular localization of Spargel/dPGC-1 occurs independent of Insulin signaling.

## Spargel/dPGC-1 doesn't influence antioxidant enzymes

The apparent involvement of PGC-1 in oxidative metabolism has been established from the following: PGC-1 activates Nuclear Respiratory Factor1 (*NRF1*); muscle specific overexpression of PGC-1α induces specific antioxidants like *Sod2* and *GpX* transcription activity (St-Pierre et al., 2006) whereas ablation of PGC-1α in cultured cell cause down regulation of a whole panel of antioxidants including *SOD1, SOD2, GpX*, *UCP1*, and *UCP2*, resulting in hypersensitivity to hydrogen peroxide induced oxidative stress (St-Pierre et al., 2006). Another interesting observation is that cells from patients with Friedrich's Ataxia show coordinated suppression of PGC-1 and antioxidant enzymes (Coppola et al., 2009). It was inferred from these observations that PGC-1 controls mitochondrial reactive oxygen species (ROS) by regulating the antioxidant defense system (Austin and St-Pierre, 2012).

**Figure 2:**
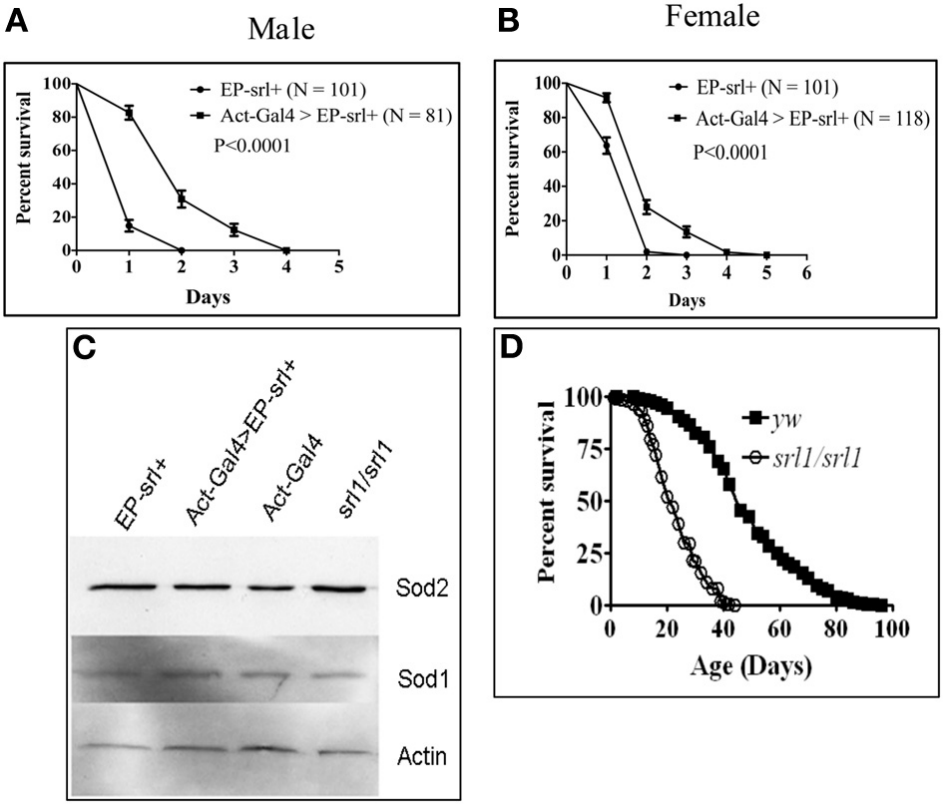
**Spargel gain of function was achieved by activating an *EP*-*srl*^+^ transgene ubiquitously with the help of *Actin-GAL4* driver (see Kapahi et al., 2004, for detail)**. Percent survival was measured following exposure to 20 mM paraquat made in 1% sucrose solution. **(A,B)** Male and female flies overexpressing Spargel/dPGC-1 survive longer in paraquat than the control indicating that Spargel/dPGC-1 overexpression cause increased resistance to oxidative stress, **(C)** Spargel/dPGC-1 overexpression or its ablation in *srl^1^* does not alter the expression of SOD2 and SOD1 antioxidant enzymes, **(D)** Reduced Spargel/dPGC-1 in *srl^1^* cause significant reduction in life span.

We attempted to validate this prediction in a whole animals model by utilizing the *Drosophila spargel* mutant hypomorph, *srl^1^* (Tiefenbock et al., 2010) and a *spargel* transgenic line that is capable of overexpressing Spargel/dPGC-1 (Mukherjee and Duttaroy, 2013). For systemic oxidative stress generation, we used methyl violgen (commercially known as “paraquat”) that is stable at room temperature. Male and female flies overexpressing Spargel were exposed to 20 mM paraquat and both survived better than the control (Figures 2A,B). After 48 h of paraquat treatment, Spargel/dPGC-1 overexpression helped 50% males to remain viable, where as in control flies 50% survival was attained within 24 h. Female flies appear slightly more sensitive to paraquat treatment although a significant difference still persists with respect to the control (Figures 2A,B). This increased resistance to oxidative stress in Spargel/dPGC-1 overexpressing flies makes it tempting to conclude that Spargel/dPGC-1 is also involved in oxidative stress resistance and imposes the same effect on antioxidant defense enzymes like PGC-1. However, this expectation may not be true since the expression of two front line antioxidant defense enzymes SOD2 and SOD1 remain unchanged when Spargel/dPGC-1 is overexpressed (gain of function) or reduced in *srl^1^* hypomorphs (Figure 2C). Since these experiments were performed on whole animals as opposed to cultured cells, it might be necessary to check the status of these antioxidant enzymes in *PGC-1KO* mice. Secondly, since it is involved in the Insulin-TOR signaling (Mukherjee and Duttaroy, 2013), Spargel/dPGC-1 might utilize other stress resistance pathways (which remain to be tested) such as FoxO or Jnk as opposed to antioxidant enzymes. Incidentally, a recent study in *Drosophila* showed that *Wolbachea* induced metabolic stress promotes mitogenesis through activation of Spargel/dPGC-1 (Chen et al., 2012). Clearly, more needs to be done to understand the relationship between Spargel/dPGC-1 and stress resistance.

## Growth, longevity, and aging

Insulin-TOR signaling pathway is involved in systemic regulation of growth, longevity and aging in all taxa (Hietakangas and Cohen, 2009; Partridge et al., 2011). In the absence of Insulin, PGC-1 expression is elevated in liver and gluconeogenesis is initiated (Puigserver et al., 2003). Therefore liver-specific knock down of PGC-1 shows higher insulin sensitivity in mice (Koo et al., 2004). On the contrary, absence of PGC-1 in skeletal muscles imposes no effect on insulin sensitivity (Zechner et al., 2010). Thus one can assume that the metabolic role of PGC-1 and its relationship with Insulin/TOR signaling became more tissue specific during the course of evolution.

The requirement of Spargel/dPGC-1 in the Insulin-TOR signaling pathway (Tiefenbock et al., 2010; Mukherjee and Duttaroy, 2013) automatically connects it to the cell growth process. Spargel/dPGC-1 is a terminal effector of this pathway hence reduced *spargel* expression results in growth retardation with smaller body size and developmental delays, though Spargel/dPGC-1 overexpression has no immediate effect on growth (Rera et al., 2011; Mukherjee and Duttaroy, 2013).

With respect to longevity, members of the Insulin-TOR signaling pathway extend life span in *C. elegans* (Vellai et al., 2003; Kenyon, 2010; Partridge et al., 2011), *Drosophila* (Clancy et al., 2001; Tatar et al., 2001; Kapahi et al., 2004) and mice (Fontana et al., 2010; Kenyon, 2010) by delaying the aging process. Mutant forms of *Insulin receptor (InR)*, the Insulin receptor substrate *chico* and TOR all extend longevity, whereas for *FoxO* lifespan extension happens through its overexpression. Spargel/dPGC-1's action on longevity is apparent as reduced Spargel/dPGC-1 expression cause significant shortening of life span (Figure 2D). Reduced ROS production and stem cell over proliferation were cited as two major reasons for Spargel/dPGC-1 mediated extension of lifespan (Rera et al., 2011), though the gain of function effects are specific to the intestinal stem cells as opposed to the Insulin and TOR mediated lifespan extensions, which are largely ubiquitous. So, research on *Drosophila* is pioneering for understanding the aging aspects of the PGC-1 group of proteins, and we are anxiously awaiting studies on how the mammalian PGC-1 protein influences aging.

To summarize, the effects of Spargel/dPGC-1 on growth and survival are positive, like all the other members of the Insulin/TOR signaling pathway (Mukherjee and Duttaroy, 2013). So, *spargel/dPGC-1* loss of function is lethal (Duttaroy et al., in preparation), *spargel/dPGC-1* hypomorphs have a much shorter life span (Figure 2D) where as ubiquitous gain of Spargel/dPGC-1 function does not extend lifespan (Rera et al., 2011). Thus, Spargel/dPGC-1 completes the function of Insulin-TOR pathway leading to survival.

## Is Spargel/dPGC-1 essential for female fertility?

**Figure 3:**
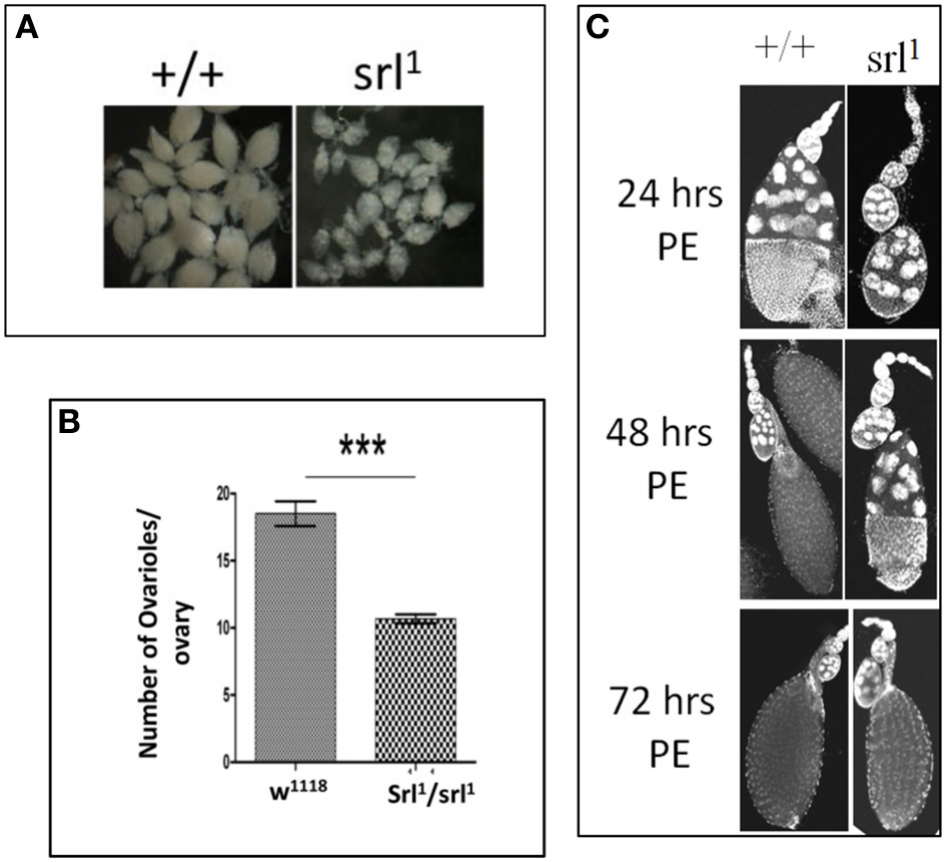
**Possible requirement of Spargel/dPGC-1 during oogenesis. (A)** Mature ovaries from *spargel* hypomorph *srl^1^* females are much smaller in size with respect to the control ovaries of same age, **(B)** Quantification of number of ovarioles suggest *srl^1^* ovaries carry about 40% less number of ovarioles with respect to the wild type ovaries. **(C)** Time course analysis of oogenesis shows *srl^1^* ovaries matures more slowly in comparison to the wild type ovaries (PE, post eclosion). ^***^P < 0.001.

Reduced Spargel/dPGC-1 expression causes only a few viable adults to appear from *srl^1^* homozygous mothers, indicating that fecundity is seriously compromised in *srl^1^* hypomorphic females though the fertility of *srl^1^* males remain unchanged (Table 1). The growth retardation effect of *srl^1^* could be the reason for the appearance of dysgenic ovaries, which carry about half the number of ovarioles compared to the wild type (Figures 3A,B). A simple time course analysis of oogenesis revealed that *srl^1^* ovaries develop slowly. In 24 h post eclosion (PE) wild type ovarioles reach up to stage 10/11 whereas ovarioles in *srl^1^* are lagging behind in stage 6/7. By 48 h mature oocytes appear in the wild type ovarioles, whereas most *srl^1^* ovarioles are observed around stage 10 during this time (Figure 3C). Efforts are underway to pin down the requirement of Spargel/dPGC-1 during oogenesis through its selective ablation in the ovaries. Interestingly, decreased female fecundity results from oogenesis defects in *InR* and *chico* mutants (Tatar et al., 2001; Partridge et al., 2011). These observations suggested a correlation between Insulin signaling and reproductive aging. Incidentally, the effect of *srl^1^* mutant on female fecundity and oogenesis appears to suggest that Spargel could be important for reproductive aging.

**Table 1:** **Fertility of *srl^1^* females**.

**Genotype**	**Fecundity (# of adults)**
y w	361
*y w; srl^1^* female X *y w* male	8
*y w; srl^1^* male X *y w* female	296
*y w; srl^1^* female X *y w, srl^1^* male	0

**Figure 4:**
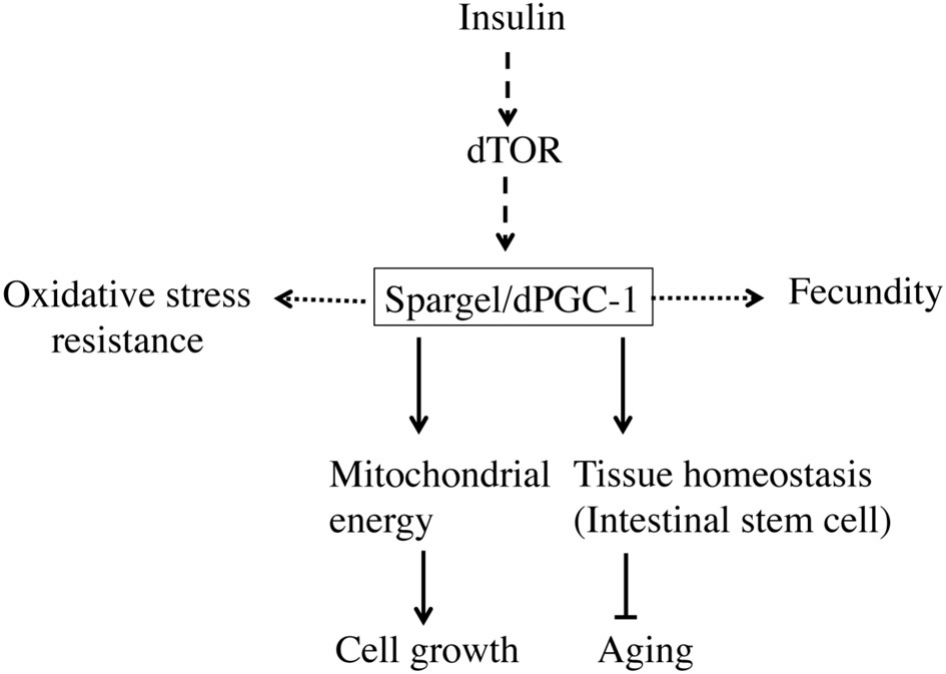
**Spargel/dPGC-1 plays a central role in maintaining homeostasis**. Spargel/dPGC-1 is acting downstream of Insulin/TOR signaling pathway. At this position, Spargel/dPGC-1 is regulating the mitochondrial energy flow into the system leading to cell growth. In addition, Spargel/dPGC-1 provides oxidative stress resistance. Though unlike mammals, this stress resistance does not confer SOD1 and SOD2 protein expression. Spargel/dPGC-1 also affects *Drosophila* fecundity possibly through oogenesis. Moreover, Spargel/dPGC-1 regulates tissue homeostasis by controlling over proliferation of intestinal stem cells in older flies.

In summary, the PGC-1 group of proteins retained many important biological functions between vertebrates and invertebrates, though many are still unknown. The overarching hypothesis of this article is that Spargel/dPGC-1 can pave the way to uncover newer biological functions, which can be tested in mammalian PGC-1 (Figure 4). Given the amount of functional overlaps already existing between PGC-1 and Spargel/dPGC-1, some of these similarities may be of immediate interest, including the role of the PGC-1 group of proteins on transcription and RNA processing and finding PGC-1 interacting proteins involved in growth and metabolism. Available genetic tools and genomic reagents in *Drosophila* should come in handy for exploring the functionality of this omnipotent transcription co-activator.

### Conflict of interest statement

The authors declare that the research was conducted in the absence of any commercial or financial relationships that could be construed as a potential conflict of interest.

## References

[B1] Austin S. St-Pierre J. (2012). PGC1alpha and mitochondrial metabolism–emerging concepts and relevance in ageing and neurodegenerative disorders. J. Cell Sci. 125, 4963–4971 10.1242/jcs.113662 23277535

[B2] Bugger H. Guzman C. Zechner C. Palmeri M. Russell K. S. Russell R. R. 3rd. (2011). Uncoupling protein downregulation in doxorubicin-induced heart failure improves mitochondrial coupling but increases reactive oxygen species generation. Cancer Chemother. Pharmacol. 67, 1381–1388 10.1007/s00280-010-1441-7 20809120 PMC3051028

[B3] Chen S. Oliveira M. T. Sanz A. Kemppainen E. Fukuoh A. Schlicht B. (2012). A cytoplasmic suppressor of a nuclear mutation affecting mitochondrial functions in Drosophila. Genetics 192, 483–493 10.1534/genetics.112.143719 22851652 PMC3454878

[B4] Clancy D. J. Gems D. Harshman L. G. Oldham S. Stocker H. Hafen E. (2001). Extension of life-span by loss of CHICO, a Drosophila insulin receptor substrate protein. Science 292, 104–106 10.1126/science.1057991 11292874

[B5] Coppola G. Marmolino D. Lu D. Wang Q. Cnop M. Rai M. (2009). Functional genomic analysis of frataxin deficiency reveals tissue-specific alterations and identifies the PPARgamma pathway as a therapeutic target in Friedreich's ataxia. Hum. Mol. Genet. 18, 2452–2461 10.1093/hmg/ddp183 19376812 PMC2694693

[B6] Fontana L. Partridge L. Longo V. D. (2010). Extending healthy life span–from yeast to humans. Science 328, 321–326 10.1126/science.1172539 20395504 PMC3607354

[B7] Gershman B. Puig O. Hang L. Peitzsch R. M. Tatar M. Garofalo R. S. (2007). High-resolution dynamics of the transcriptional response to nutrition in Drosophila: a key role for dFOXO. Physiol. Genomics 29, 24–34 10.1152/physiolgenomics.00061.2006 17090700

[B8] Handschin C. Spiegelman B. M. (2006). Peroxisome proliferator-activated receptor gamma coactivator 1 coactivators, energy homeostasis, and metabolism. Endocr. Rev. 27, 728–735 10.1210/er.2006-0037 17018837

[B9] Hietakangas V. Cohen S. M. (2009). Regulation of tissue growth through nutrient sensing. Annu. Rev. Genet. 43, 389–410 10.1146/annurev-genet-102108-134815 19694515

[B10] Kapahi P. Zid B. M. Harper T. Koslover D. Sapin V. Benzer S. (2004). Regulation of lifespan in Drosophila by modulation of genes in the TOR signaling pathway. Curr. Biol. 14, 885–890 10.1016/j.cub.2004.03.059 15186745 PMC2754830

[B11] Kenyon C. J. (2010). The genetics of ageing. Nature 464, 504–512 10.1038/nature08980 20336132

[B12] Koo S. H. Satoh H. Herzig S. Lee C. H. Hedrick S. Kulkarni R. (2004). PGC-1 promotes insulin resistance in liver through PPAR-alpha-dependent induction of TRB-3. Nat. Med. 10, 530–534 10.1038/nm1044 15107844

[B13] Lin J. Handschin C. Spiegelman B. M. (2005). Metabolic control through the PGC-1 family of transcription coactivators. Cell Metab. 1, 361–370 10.1016/j.cmet.2005.05.004 16054085

[B14] Liu C. Lin J. D. (2011). PGC-1 coactivators in the control of energy metabolism. Acta Biochim. Biophys. Sin. (Shanghai) 43, 248–257 10.1093/abbs/gmr007 21325336 PMC3063079

[B15] Matsuda S. Harries J. C. Viskaduraki M. Troke P. J. Kindle K. B. Ryan C. (2004). A conserved alpha-helical motif mediates the binding of diverse nuclear proteins to the SRC1 interaction domain of CBP. J. Biol. Chem. 279, 14055–14064 10.1074/jbc.M310188200 14722092

[B16] Monsalve M. Wu Z. Adelmant G. Puigserver P. Fan M. Spiegelman B. M. (2000). Direct coupling of transcription and mRNA processing through the thermogenic coactivator PGC-1. Mol. Cell 6, 307–316 10.1016/S1097-2765(00)00031-9 10983978

[B17] Mukherjee S. Duttaroy A. (2013). Spargel/dPGC-1 is a new downstream effector in the insulin-TOR signaling pathway in Drosophila. Genetics 195, 433–441 10.1534/genetics.113.154583 23934892 PMC3781971

[B18] Partridge L. Alic N. Bjedov I. Piper M. D. (2011). Ageing in Drosophila: the role of the insulin/Igf and TOR signalling network. Exp. Gerontol. 46, 376–381 10.1016/j.exger.2010.09.003 20849947 PMC3087113

[B19] Puigserver P. Rhee J. Donovan J. Walkey C. J. Yoon J. C. Oriente F. (2003). Insulin-regulated hepatic gluconeogenesis through FOXO1-PGC-1alpha interaction. Nature 423, 550–555 10.1038/nature01667 12754525

[B20] Puigserver P. Spiegelman B. M. (2003). Peroxisome proliferator-activated receptor-gamma coactivator 1 alpha (PGC-1 alpha): transcriptional coactivator and metabolic regulator. Endocr. Rev. 24, 78–90 10.1210/er.2002-0012 12588810

[B21] Puigserver P. Wu Z. Park C. W. Graves R. Wright M. Spiegelman B. M. (1998). A cold-inducible coactivator of nuclear receptors linked to adaptive thermogenesis. Cell 92, 829–839 10.1016/S0092-8674(00)81410-5 9529258

[B22] Rera M. Bahadorani S. Cho J. Koehler C. L. Ulgherait M. Hur J. H. (2011). Modulation of longevity and tissue homeostasis by the Drosophila PGC-1 homolog. Cell Metab. 14, 623–634 10.1016/j.cmet.2011.09.013 22055505 PMC3238792

[B23] St-Pierre J. Drori S. Uldry M. Silvaggi J. M. Rhee J. Jager S. (2006). Suppression of reactive oxygen species and neurodegeneration by the PGC-1 transcriptional coactivators. Cell 127, 397–408 10.1016/j.cell.2006.09.024 17055439

[B24] Takano A. Usui I. Haruta T. Kawahara J. Uno T. Iwata M. (2001). Mammalian target of rapamycin pathway regulates insulin signaling via subcellular redistribution of insulin receptor substrate 1 and integrates nutritional signals and metabolic signals of insulin. Mol. Cell. Biol. 21, 5050–5062 10.1128/MCB.21.15.5050-5062.2001 11438661 PMC87231

[B25] Tatar M. Kopelman A. Epstein D. Tu M. P. Yin C. M. Garofalo R. S. (2001). A mutant Drosophila insulin receptor homolog that extends life-span and impairs neuroendocrine function. Science 292, 107–110 10.1126/science.1057987 11292875

[B26] Tiefenbock S. K. Baltzer C. Egli N. A. Frei C. (2010). The Drosophila PGC-1 homologue Spargel coordinates mitochondrial activity to insulin signalling. EMBO J. 29, 171–183 10.1038/emboj.2009.330 19910925 PMC2808377

[B27] van der Linde K. Houle D. (2008). A supertree analysis and literature review of the genus Drosophila and closely related genera (Diptera, Drosophilidae). Insect. Syst. Evol. 39, 241–267 10.1163/187631208788784237

[B28] Vellai T. Takacs-Vellai K. Zhang Y. Kovacs A. L. Orosz L. Muller F. (2003). Genetics: influence of TOR kinase on lifespan in *C. elegans*. Nature 426, 620 10.1038/426620a 14668850

[B29] Wang J. Li Y. Zhang M. Liu Z. Wu C. Yuan H. (2007). A zinc finger HIT domain-containing protein, ZNHIT-1, interacts with orphan nuclear hormone receptor Rev-erbbeta and removes Rev-erbbeta-induced inhibition of apoCIII transcription. FEBS J. 274, 5370–5381 10.1111/j.1742-4658.2007.06062.x 17892483

[B30] Zechner C. Lai L. Zechner J. F. Geng T. Yan Z. Rumsey J. W. (2010). Total skeletal muscle PGC-1 deficiency uncouples mitochondrial derangements from fiber type determination and insulin sensitivity. Cell Metab. 12, 633–642 10.1016/j.cmet.2010.11.008 21109195 PMC2999961

